# The Role of Psychosocial Stress on a Family-Based Treatment for Adolescents with Problematic Behaviors

**DOI:** 10.3390/ijerph15091867

**Published:** 2018-08-29

**Authors:** Jesús Maya, Bárbara Lorence, Victoria Hidalgo, Lucía Jiménez

**Affiliations:** 1 Department of Developmental and Educational Psychology, University of Seville, Camilo José Cela s/n, 41018 Seville, Spain; jmaya3@us.es (J.M.); victoria@us.es (V.H.); luciajimenez@us.es (L.J.); 2 Department of Social, Developmental and Educational Psychology, University of Huelva, Avda. Tres de Marzo s/n, 21071 Huelva, Spain

**Keywords:** adolescence, behavior problems, stressful life events, emotional intelligence, parent attachment, effectiveness, family therapy

## Abstract

The stressful life events experienced by adolescents with problematic behaviors, should be considered for implementing effective interventions. This study aimed to examine the adjustment of adolescents with problematic behaviors, and to assess the effectiveness of a family-based treatment, namely Scene-Based Psychodramatic Family Therapy (SB-PFT), according to different stress profiles. Ten SB-PFT sessions, over 17 trials were implemented. Stressful life events and adolescent adjustment were evaluated at pretest and posttest, for the SB-PFT participants (*n* = 104 adolescents) and a control group (*n* = 106). The adolescents were categorized into three profiles depending on the nature of the stressors: family stress profile, individual and family stress profile, and low stress profile. The individual and family stress group showed worse adjustment. Effectiveness analyses revealed improvements in SB-PFT participants’ emotional intelligence, but not in anger and hostility. Furthermore, adolescents with low and family-related stress profiles showed enhancements in parent attachment. In conclusion, interventions involving adolescents with problematic behaviors must be tailored to the stressful life events experienced. Specific treatments should be used alongside SB-PFT, when adolescents are met with individual-related stress. Nevertheless, SB-PFT seemed to promote emotional intelligence and parent attachment, particularly in adolescents with problematic behaviors that experienced only family stressors.

## 1. Introduction

Adolescents deal with several biological, cognitive, and psychosocial changes [1], some of which may be identified as potentially stressful among this population [2]. According to Lazarus and Folkman [3], how the individuals assess potentially stressful events and the resources they have to address these events will determine their stress status. Psychosocial stress in adolescents is accentuated by the presence of stressors or stressful life events that threaten their capabilities [2]. These stressful events may vary depending on the specific life domain, and can compromise adolescent development and well-being [2,4].

Special attention has been paid to the impact of stressful life events of an environmental nature on adolescence, such as living in priority neighborhoods that cope with social and economic challenges [2,5]. The effects of family stress factors, such as low household incomes, parental conflict and divorce, and a parent’s illness have been also examined [6,7]. The presence of these environmental and family stressors is negatively related to adjustment and mental health in adolescents, and precipitates the onset of problematic behaviors [2,6,8]. Specifically, financial instability and belonging to a priority area are associated with increased psychosocial stress and maladjustment in adolescence [2,9]. Family stressful events, such as marital conflict and parental divorce, also represent situations which significantly increase an adolescent’s family stress load, and are related to the presence of problematic behaviors [6,7]. Furthermore, these stressful events may be comorbid. The negative effects of divorce on children have been shown to seemingly increase when families face economic hardship [7]. Along these lines, another source of family stress, such as parental illness, also seems to correlate with the presence of problematic behaviors during adolescence [6].

These environmental and family stressors can be accompanied by individual stressful events experienced by adolescents themselves, which place these youths in a particularly vulnerable situation [10,11]. Specifically, exposure to serious situations, such as peer victimization or bullying, family abuse, or sexual abuse has a negative impact on adolescent adjustment and mental health [12]. Of these individual stressors, peer victimization or bullying is reported as being the most prevalent among adolescents [13,14]. Adolescents’ perceptions about feeling rejected, alienated, and harassed by peers during the bullying process can have major psychological consequences such as anxiety, depression, and externalizing problems as aggressive and disruptive behaviors [15,16,17,18]. Additionally, these symptoms in extreme cases during early adolescence can lead to a bipolar disorder diagnosis. Bipolar disorder may even be accompanied by abnormalities in white matter connectivity, and impairment of the neuronal circuits involved in emotional regulation [19]. Moreover, there are other contexts in which adolescents may also suffer from violence, such as the family context. These situations may be a leading source of psychosocial stress for adolescents. Specifically, aggressive behaviors from parents towards their sons and daughters are related to adolescent maladjustment and problematic behaviors [20,21]. Finally, although epidemiologically less frequent [13], when referring to individual stressful life events in young people, we cannot ignore cases of sexual abuse. Specifically, these events can precipitate the development of internalizing problems, such as depression [22] and problematic behaviors including sexual offending and violent acts [23,24].

The link between stressful life events and the problematic behaviors presented above, could be explained from several theories. General strain theory (GST) [25] highlights how exposure to stressful events generates negative emotions, such as anger and hostility, which hinder the individual’s ability to successfully respond to environmental demands, instead promoting violent behavior as a way of escaping from stressors. The frustration–aggression hypothesis [26], argues that stressful events perceived as uncontrollable and unfair bring with them a series of negative feelings of frustration, thereby increasing the individual’s risk for developing problematic behaviors. More recently, I^3^ theory [27,28] suggests that tackling stressful events uses up the individual’s self-regulation strategies, thus making it difficult to develop the skills needed to inhibit any violent impulses. In sum, stressful life events mobilize the resources that adolescents draw on to manage stressors; this increases levels of emotional strain and facilitates the emergence of problematic behaviors in the absence of adequate cognitive, behavioral, and emotional stressor management.

Although stressful life events hinder adolescent development and a smooth transition to adulthood, not all stressful situations have the same consequences for adolescents [29,30]. Thus, the presence of protective factors at family (e.g., a strong parental bond) and individual level (such as good emotional intelligence), can buffer the negative impact of stressful life events [29,31]. In terms of the parent–child relationship, it has been shown that positive parenting practices reduce the negative psychological consequences behind stressful life events [32,33]. Thus, positive parenting is associated with decreased levels of psychosocial stress and better self-regulation in adolescents, so they can better cope with stressful situations [34,35]. Furthermore, we count on evidence that a parent–adolescent relationship built on support, communication, and caring renders adolescents less likely to develop problematic behaviors when faced with individual stressful situations, such as peer victimization [17,36,37]. Regarding individual protective factors, emotional intelligence in adolescents appears to moderate the negative impact of perceived psychosocial stress [38,39]. Moreover, emotional understanding and emotional management mitigates the negative effects of perceived psychosocial stress and enhances life satisfaction in adolescence [31,40]. In fact, Hsieh et al. [41] observed that a better understanding of own and others’ emotions in adolescent victims of peer victimization moderated their degree of hostility.

### 1.1. Interventions Involving Adolescents with Problematic Behaviors. The Scene-Based Psychodramatic Family Therapy

Throughout this introduction, we have described how the stressful life events of different nature that adolescents face, represent a risk factor for the onset of adolescent’s problematic behaviors [6,8,42]. Problematic behaviors exhibited by adolescents, such as disobedience, fighting, lying, physical and/or verbal aggression, expulsion from school, and other forms of maladaptive conduct constitute nowadays a social concern, as may culminate with young boys and girls being involved in the criminal justice system [43,44].

Recent decades have seen a significant increase in interventions aimed at reducing disruptive behaviors in adolescents exposed to stressful situations. These initiatives seek to promote individual, family, and environmental protective factors to buffer the negative effects of exposure to risky situations that compromise adolescent well-being, i.e., neighborhoods with high rates of violence, truancy, family stress, peer substance abuse, etcetera [12,29,45].

Adolescents with problematic behaviors are often described homogeneously, although diverse risk profiles emerge in this population, both at an individual and family level [44]. For this reason, diverse profiles of adolescents with problematic behaviors should be considered when designing and evaluating intervention programs aimed at this population, to diversify and adapt available resources according to their specific profiles [46]. Specifically, the empirical evidence available underlines the importance of controlling for the presence and nature of stressful events to ensure that these interventions are effective [10,11].

Current family-based treatment programs for adolescents with behavior problems and their parents, recognized by Blueprints for healthy youth development [47], include Multisystemic Therapy (MST) [48] and Functional Family Therapy (FFT) [44]. Both treatments are based on the family therapy model, and aim to reduce adolescent problematic behaviors. The outcomes that these treatments seek to achieve include promoting positive parenting, positive adaptation and adjustment in adolescents, the acquisition of conflict resolution strategies, and improving family relationships as a main protective factor [49,50].

The Scene-Based Psychodramatic Family Therapy (SB-PFT) constitutes a novel treatment, specifically designed for families with adolescents with problematic behaviors from priority neighborhoods [51,52]. SB-PFT is a specific approach aimed at improving family relationships and reducing adolescent’s problematic behaviors [51,52,53]. It integrates the principles and techniques of systemic family therapy (e.g., the importance of including all family members and focusing intervention on dysfunctional family relationships), with psychodramatic techniques (e.g., role-playing, role reversal, mirror, scene) using a multiple-family group format [52,54,55]. The usefulness of using psychodramatic techniques in the therapeutic process has recently been recognized in the European context [55]. From the perspective of multiple-family groups followed by SB-PFT, several family systems are worked on together therapeutically [56]. Multiple-family groups have proven effective in promoting parent-child communication and problem solving [56]. In SB-PFT, 10 weekly 2-h intervention sessions are held [51,52]. Each SB-PFT group is led by two psychotherapists trained in systemic family therapy and psychodrama, and two auxiliary egos who are experts in psychodramatic techniques [52]. The core contents dealt with during the sessions cover: conflicts in parent–adolescent interaction, and adolescent-experienced stressful situations (such as parental separation and conflict, violence at home, and peer conflict). The contents targeted in each SB-PFT session depend on the needs of the participants assessed by the professionals (i.e., adolescents’ conflicts with their parents or in the social context), as well as on the problem situations that the participants express during the therapeutic process. Each session of the SB-PFT follows a similar structure [52]. First, the therapists and auxiliary egos decide on the therapeutic objectives of the session, according to the components of the family system which may be influencing the problematic behaviors of the adolescents. Second, the auxiliary egos dramatize a conflictive situation. Finally, adolescents and parents discuss the content, and engage in a role-play with the auxiliary ego to express their emotions, become aware of their strengths and difficulties as a family, be able to order their emotions, and develop other ways to resolve family conflicts (or social conflicts) without using disruptive behaviors [52]. In this process, the psychodramatic techniques and the group therapeutic factors [55,57] play a relevant role. Specifically, the importance of knowing other adolescents in the same situation and acknowledging interpersonal learning are important components of the SB-PFT. In general terms, the SB-PFT seeks to provide an opportunity for adolescents and their parents to not only express and free the emotional strain behind existing conflicts, but to also acquire other conflict resolution strategies through dramatization and group integration in a therapeutic context with some family system [55,58].

### 1.2. Present Study

Although SB-PFT constitutes an innovative treatment, evidence supporting its effectiveness has been found in relation to emotional intelligence, parent attachment, and antisocial behavior [55]. To our knowledge, there are no studies that describe and assess the role of psychosocial stress on adolescents participating in SB-PFT, with a focus on the nature of the stressors.

Data on the high prevalence of stressful life events among Spanish adolescents (83% have experienced a stressful situation in their lives), have driven the development of initiatives and interventions involving these adolescents [13]. This high prevalence suggests the need for exploring in more depth, the role that psychosocial stress plays during adolescence, and for developing evidence-based interventions that minimize the negative effects of specific stressful life events. Hence, the differential impact of stressful life events, according to specific life domains, should be considered [4]. For this reason, in this study the adolescents were grouped according to the presence of family or/and individual stressors.

Specifically, this study aimed to: (1) Examine variability in emotional intelligence, aggressive behavior, and parent attachment in adolescents with problematic behaviors, according to the experience of stressful life events; and (2) Assess the effectiveness of SB-PFT treatment in adolescents with problematic behaviors, by each stress profile. Concerning the first objective, we expect that adolescents who had experienced both family and individual stressors, will exhibit the highest maladaptive profile. Regarding the second objective, we expect SB-PFT effectiveness for all the stress profiles, compared to the control group. Specifically, because SB-PFT is a multiple-family intervention, we expect higher effectiveness for adolescents who had experienced family stressors.

## 2. Materials and Methods

### 2.1. Study Design

This study was part of a larger research project aimed at describing and evaluating the effectiveness of a scene-based psychodramatic family therapy (SB-PFT) treatment run by Child Welfare Services (CWS), across 10 priority areas of southern Spain from 2015 to 2017. CWS are public services that deliver assistance to mitigate family problem situations. Their intervention areas cover environmental changes, such as improving family functioning, to ensure comprehensive child and adolescent development [59]. For this study, a quasi-experimental design was followed, using pretest (T1) and posttest (T2) measures, for both the intervention (IG) and control group (CG).

### 2.2. Participants

**Figure 1:**
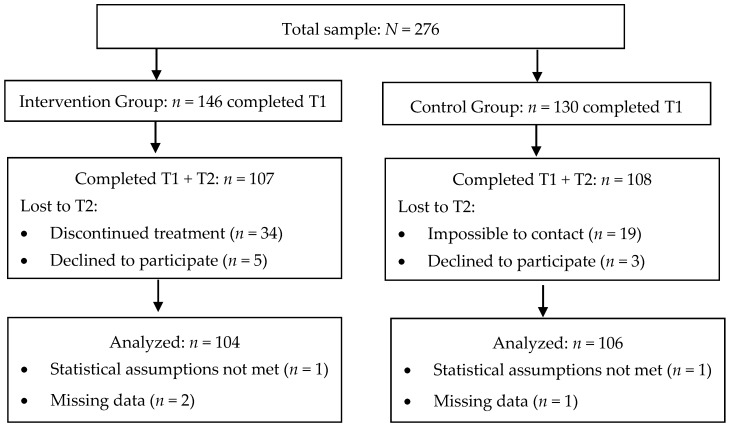
Flowchart of participants throughout the study.

For the SB-PFT treatment, 17 trials were conducted with 10 sessions per trial (one session per week), and an average of 8 adolescents and their caregivers per trial. For the purpose of this study, 210 adolescents participated. Each adolescent from the IG participated in only one trial. Participants attended, on average, 8 sessions. The flow of cases through the study is shown in Figure 1. As can be observed in this figure, a 28.77% IG dropout rate and an 18.46% CG dropout rate were obtained. The IG ended up comprising 104 adolescents, and the CG 106 adolescents.

The baseline characteristics of the IG completers, the CG completers, and the total sample are shown in Table 1. Regarding sociodemographic characteristics, a comparable profile was observed between the IG and the CG across all variables examined. As can be seen in Table 1, the sample was equally distributed by gender, yielding an average age of 14 years. By studying the psychosocial stress profiles, severe financial problems and chronic parental conflict, emerged as the most salient family stress factors. Individual stress factors were less frequent, with bulling or peer victimization ranked as the most prevalent stressors. Stressful event occurrence was comparable between the IG and the CG, except for chronic parental conflict, parent’s new partner, and parent’s mental or physical illness (higher occurrence reported in the IG).

Based on the stressful life events reported in this table, four stress profiles were established: (1) a family stress profile (FSP), comprising participants that had experienced only family-related stress factors (*n* = 92; 43.80% of the total sample); (2) an individual stress profile (ISP), made up of those adolescents who only experienced individual stress factors associated with violent episodes (*n* = 10; 4.8% of the sample); (3) an individual and family stress profile (IFSP), including those participants who experienced both family and individual stress factors (*n* = 71; 33.80% of the sample); and (4) a low stress profile (LSP), comprising adolescents who did not experience any family or individual factors in the five years prior to the study, from those listed in Table 1 (*n* = 37; 17.60% of the total sample), despite residing in potentially stressful neighborhoods. Given profile 2 (ISP) was made up of a small number of participants (*n* = 10), this group was excluded from subsequent analyses due to statistical power concerns. Stress profiles were distributed throughout both the IG and the CG, as follows: FSP (IG: 52.17%, CG: 47.83%); IFSP (IG: 59.15%, CG: 40.84%); and LSP (IG: 27.03%, CG: 72.97%).

### 2.3. Measures

Sociodemographic profile: This questionnaire was developed ad hoc by the researchers to collect from adolescents’, sociodemographic information on individual variables and family characteristics through six items: sex, age, academic year, family structure, primary caregiver at home, and number of family members.

Stressful life events Inventory [60]: An adaptation of the Spanish questionnaire on stressful life events was used to assess psychosocial stress profiles [60]. Eight stressful life events were intentionally selected for this project: family-related stressful events (parents’ divorce, chronic parental conflict, parent’s new partner, severe financial problems, and parent’s mental or physical illness); and individual stressful events (peer victimization, intra-family violence, sexual harassment or abuse). A 5-year period was considered for the occurrence of the stressors, rated in a dichotomist scale (0 = no, 1 = yes).

Emotional Quotient Inventory—Youth Version [61]: This 60-item questionnaire measures five components of emotional intelligence: intrapersonal (e.g., “It’s easy to tell people how I feel”); interpersonal (e.g., “I know when people are upset, even when they say nothing”); adaptability (e.g., “I can understand hard questions”); stress management (e.g., “I get angry easily”); and general mood (e.g., “I am happy”). Adolescents were required to rate the degree to which each item was true for them on a 4-point scale (from 1 = Very seldom true or not true of me to 4 = Very often true of me or true of me). The reliability coefficients computed in this study were listed for each scale: intrapersonal (α_T1_ = 0.61 and α_T2_ = 0.70), interpersonal (α_T1_ = 0.77 and α_T2_ = 0.79), adaptability (α_T1_ = 0.80 and α_T2_ = 0.85), stress management (α_T1_ = 0.83 and α_T2_ = 0.82), and general mood (α_T1_ = 0.89 and α_T2_ = 0.86).

The Inventory of Parent and Peer Attachment [62]: The parent attachment scales of this measure were included in the present study, totaling 25 items related to communication (e.g., “My mother/father helps me to talk about my difficulties”); trust (e.g., “I feel my mother/father does a good job as my parent”); and alienation (e.g., “I feel angry with my mother/father”) between the adolescent and his/her primary caregiver. Each item was rated on a 5-point scale (from 1 = Almost never or never true to 5 = Almost always or always true). The reliability coefficients in this study were described as: communication (α_T1_ = 0.83 and α_T2_ = 0.84), trust (α_T1_ = 0.87 and α_T2_ = 0.86), and alienation (α_T1_ = 0.61 and α_T2_ = 0.67).

Aggression Questionnaire [63]: Two dimensions from the 29-item version, adapted by Santiesteban and Alvarado [64], were used in this study to assess anger (e.g., “I have trouble controlling my temper”), and hostility (e.g., “I sometimes feel that people are laughing at me behind my back”). Each item was rated by the adolescents on a 5-point scale (from 1 = Extremely uncharacteristic of me to 5 = Extremely characteristic of me). The reliability coefficients founded in this study were α_T1_ = 0.64/α_T2_ = 0.69 for anger, and α_T1_ = 0.60/α_T2_ = 0.68 for hostility.

### 2.4. Procedure

CWS practitioners referred the adolescents for SB-PFT, if they met the following criteria: (a) aged between 11 and 17 years; (b) problematic behaviors, such as frequent fights with peers, alcohol use, social conflict, or expulsion from school; (c) significant impairment in family relations and a family crisis situation; and (d) consent to multiple-family treatment from both the parents and adolescents. The CG comprised a comparable sample of adolescents with problematic behaviors identified by the schools, who met the following criteria: (a) aged between 11 and 17 years; (b) residing in the same priority areas where the intervention took place; and (c) not receiving any therapeutic treatment.

The battery of questionnaires was administered by researchers at the CWS (IG) and at the schools (CG). For the IG, pretest was administered during the second SB-PFT session, and posttest took place during the last session, for those adolescents who attended at least three treatment sessions. For the CG, there was a 3-month interval between pretest and posttest assessment. All questionnaires were self-report and any doubts were clarified by the researchers. Assessment took between 20 and 45 min.

All adolescent participation was voluntary, once an informed consent form in accordance with the Declaration of Helsinki was signed. The researchers explained the aims of the project and assured participants that anonymity would be kept throughout. Ethics approval was obtained from the Andalusian Government (code 0985-M1-18).

### 2.5. Data Analyses

Analyses were performed using SPSS v.23 software (IBM, Armonk, NY, USA). The following statistical assumptions were checked, yielding satisfactory results: linearity, normality, homoscedasticity, absence of multicollinearity and singularity, and independence of residuals [65]. Two univariate outliers were detected through box-plot examination and removed from subsequent analyses [66]. Missing data at item level were examined via missing value analysis. Little’s MCAR test was used to check whether data was randomly distributed. Less than 5% of missing data were found per item, and less than 10% of items were missing per scale. Therefore, the SEM procedure was performed to impute data using the expectation-maximization (EM) algorithm from SPSS.

For inter-group comparisons, one-way ANOVAs for quantitative variables and chi-square tests for qualitative ones were performed. DMS was explored using one-way ANOVAs for paired comparisons when required. Repeated measures ANOVAs were used for longitudinal analyses, and the interaction effect time*group (IG vs. CG) was examined independently for each stress profile, as described below. The 95% confidence level was considered for the significance test. Partial eta squared and Cramer’s V were computed as effect-size indexes, respectively. Partial eta squared was deemed negligible if <0.01, small if >0.01 and <0.06, medium if >0.06 and <0.14, and large if >0.14. Cramer’s V was considered negligible if <0.10, small if >0.10 and <0.30, medium if >0.30 and <0.50, and large if >0.50 [67].

## 3. Results

### 3.1. Psychosocial Adjustment of Adolescents with Problematic Behaviors According to Their Stress Profiles

Differences in emotional intelligence, aggressive behavior, and parent attachment by stress profile were studied. The descriptive data, statistics, and effect sizes, for these analyses are shown in Table 2.

Regarding emotional intelligence variables, significant differences with small-to-medium effect sizes were observed among the three stress profiles for intrapersonal intelligence, stress management, and general mood. Specifically, DMS analyses found significant differences between IFSP adolescents and LSP adolescents, for intrapersonal intelligence, stress management, and general mood. In addition, significant differences were observed between FSP adolescents and IFSP adolescents, for intrapersonal intelligence and general mood. IFSP adolescents obtained the lowest scores across all aforementioned comparisons.

As for the aggressive behavior dimensions, significant differences with small-to-medium effect sizes were observed among the three risk stress profiles of adolescents, for both anger and hostility. DMS analyses showed that IFSP adolescents scored significantly higher in anger and hostility, compared to their LSP peers, and higher in hostility than the FSP adolescents.

Regarding parent attachment, analyses revealed significant differences in the degree of communication, trust, and alienation, yielding small-to-medium effect sizes. Specifically, DSM analyses showed that LSP adolescents obtained better scores across all parent attachment dimensions, compared to both FSP and IFSP adolescents. FSP adolescents also scored higher in alienation than their IFSP peers.

### 3.2. SB-PFT Effectiveness by Stress Profile

The effectiveness of SB-PFT, according to the stress profiles, was studied by analyzing the interaction effect time*group (IG vs. CG) using repeated measures ANOVAs separately for the LSP, the FSP, and the IFSP on the emotional intelligence, aggressive behavior, and parent attachment variables. The descriptive data, statistics, and effect sizes, for these analyses are shown in Table 3.

LSP adolescents showed significant interaction effects, with medium effect sizes, for interpersonal intelligence and parental alienation. Specifically, those adolescents who received SB-PFT achieved better results, than the adolescents who did not receive treatment.

FSP adolescents showed significant interaction effects, with small-to-medium effect sizes, for two parent attachment dimensions: communication and trust. Specifically, FSP adolescents who received SB-PFT treatment showed an improvement over the CG. The IG also showed a significant improvement in adaptability, compared to the CG (medium effect size).

Finally, for the IFSP adolescents, only general mood showed significant interaction, with a medium effect size. Specifically, the IG adolescents made greater improvements, compared to those who did not receive intervention.

**Figure 2:**
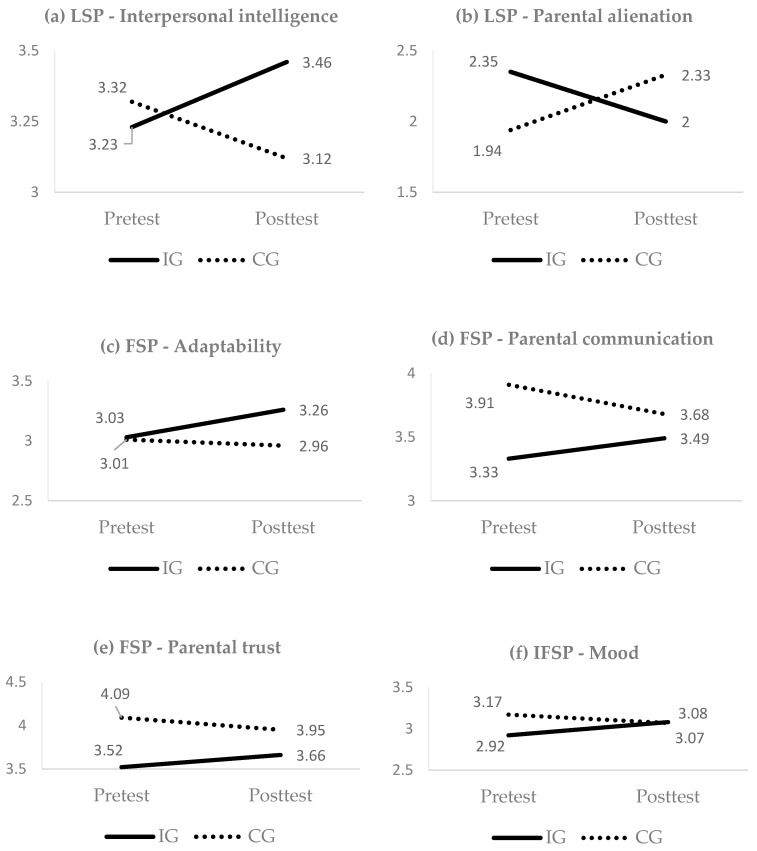
Significant interaction effects time*group (intervention vs. control). (**a**) LSP—Interpersonal intelligence: interaction effects on interpersonal intelligence in low stress profile group. (**b**) LSP—Parental alienation: interaction effects on parental alienation in low stress profile group. (**c**) FSP—Adaptability: interaction effects on adaptability in family stress profile group. (**d**) FSP—Parental communication: interaction effects on parental communication in family stress profile group. (**e**) FSP—Parental trust: interaction effects on parental trust in family stress profile group. (**f**) IFSP—Mood: interaction effects on mood in individual and family stress profile group.

The significant interaction effects described above are plotted in Figure 2.

## 4. Discussion

This study on the role of psychosocial stress in adolescents with problematic behaviors, sought to go beyond a descriptive analysis of stressful life events. Three profiles relative to the family and/or individual nature of recent stressful life events (low stress profile, LSP; family stress profile, FSP; and individual and family stress profile, IFSP), confirmed the existing diversity among adolescents growing up in priority neighborhoods. Previous studies have already reported heterogeneity among adolescents with problematic behaviors, despite them all presenting these conflictive behaviors [44]. Specifically, the profile of adolescents with family stressors (FSP) was the most prevalent, followed by adolescents having faced individual and family stressors (IFSP), and finally the group of adolescents who had not experienced any of the studied stressful life events (LSP). Furthermore, if we look at the three events of individual nature under evaluation (bullying or peer victimization, intra-family violence, and sexual abuse), we could see a consistent pattern between the present research and another Spain-based study by Pereda et al. [13], showing bullying to be the most common episode among the events that involve violence intentionally directed towards adolescent.

Regarding the first objective, the results were consistent with the researchers’ expectations. The data revealed higher maladjustment in IFSP adolescents, when compared with FSP and LSP adolescents. These findings support the need to examine the nature of stressful life events during adolescence, to enhance understanding about adjustment in this developmental period [4,29,30].

As for the differences found in aggressive behavior between the three stress profiles, the results coincided with the general strain theory [25]. The author of GST concludes that exposure to different stressful life events is linked to increased emotional strain, which in turn can lead to an increase in anger and hostility hastening the development of violent behaviors, as a way of escaping the strain brought on by stressful events. In line with other studies [10,11], adolescents who had been victims of at least one individual violent situation and that also accumulate family stressors appear to be especially vulnerable to stress that comes with high emotional strain, thus making the onset of troubled behavior more likely.

Regarding emotional intelligence, the differences observed among all three stress profiles can be explained from the perspective of I^3^ theory [27,28]. This theory holds that stressors limit the individual’s capacity for self-regulation, thus hindering their ability to inhibit violent impulses. Data collected from this study on adolescents indicated that some components of emotional intelligence, such as intrapersonal intelligence, stress management, and mood, were more affected in IFSP adolescents than in FSP and LSP peers. In line with I^3^ theory [27,28], this finding could be explained by arguing that suffering from direct violence towards oneself diminishes the adolescent’s emotional competence; in this case, intrapersonal intelligence, stress management, and mood were more restricted, which may prevent them from restraining their violent impulses.

In relation to the differences observed in parent attachment, the data once again showed greater difficulties on the part of IFSP adolescents. In this case and in contrast to aggressive behavior and emotional intelligence, the differences were also evident when comparing LSP with FSP adolescents, observing better communication, trust, and parental alienation in the LSP group. This finding supports existing evidence that highlights the protective role played by intra-family relationships, for buffering the impact of stressful life events [34].

In terms of the second objective, we partially met our expectations. Thus, adolescents participating in the SB-PFT significantly improved in some areas of emotional intelligence and parent attachment, but not significantly decreased aggressive behavior, compared to adolescents in the control group. These changes were particularly notable in the group with a family stress profile. These results confirmed the SB-PFT as a potentially effective treatment, for working in family stress situations.

First, SB-PFT showed a positive impact on mood for IFSP adolescents, compared with the CG. This finding supports those studies that highlight the importance of promoting well-being and optimism in adolescents who find themselves involved in situations, such as peer victimization, intra-family violence, and sexual abuse [14,21,22]. The lack of effectiveness of SB-PFT on other key components involving this adolescent profile (other features of emotional intelligence, aggressive behavior, or parent attachment), may be due to the seriousness of the stressful events (peer victimization, intra-family violence, or sexual abuse plus family stressors) that these boys and girls experience. There are studies that show the devastating effects of bullying, intra-family violence, and sexual abuse [15,21,23]. Thus, maybe IFSP adolescents would benefit from complementing SB-PFT with another specialized therapeutic intervention, focused on these situations marked by violence [16,24,32]. Nevertheless, SB-PFT’s effect on mood in IFSP adolescents could be considered as a necessary prerequisite for changing other well-being facets. As such, adolescents with problematic behaviors may need to start by regaining good levels of motivation and general mood, which diminished when exposed to violent situations, enabling them to then engage in a therapeutic process where they tackle other changes related to self-regulation and the attachment with their parents.

Second, the FSP adolescents that participated in SB-PFT notably showed an improvement in the parent attachment dimensions, such as communication and trust, compared to the CG. The theoretical approaches of SB-PFT based on systemic postulates [54], its methodological characteristics deriving from multiple-family group approach [56], and SB-PFT’s own objectives and content which focus primarily on improving family relationships through family conflict intervention [51,52], explain the enhanced parental trust and communication of adolescents involved in a family crisis situation. These findings coincide with previous studies on SB-PFT, where improvements in family relationships figure among the main implications of this intervention [52], and with other systemic interventions involving adolescents with problematic behaviors, such as FFT and MST [49,50]. Therefore, these results seem to confirm that SB-PFT is a potentially effective intervention to improve parent attachment, from the perspective of adolescents with a family stress profile.

Third, the LSP adolescents that benefited from SB-PFT reported positive changes, compared to the CG. Once again, an intra-family variable, such as parental alienation, was found to be a positive outcome of SB-PFT, highlighting how important it is to address family systems when behavior problems occur in adolescence [33,34]. What is more, positive changes in interpersonal intelligence among SB-PFT participants were observed, with these adolescents showing improved perspective-taking skills and a greater understanding of others’ emotions, both of which are key components when developing and assessing interventions focused on stress coping strategies [10,29]. In this case, the absence of stressful situations in personal trajectories can make adolescents more sensitive and receptive to training in emotional skills, which consider the needs of others, thus promoting a protective factor that inhibits the emergence of troubled behaviors [27,41]. In our opinion, the SB-PFT effectiveness with LSP adolescents justifies the need to conduct early interventions, which protect adolescents from the negative impact of future family and/or individual stressors [2,68].

Lastly, and most noticeably, no impact of SB-PFT on adolescents’ aggressive behavior was observed, regardless the stress profile. Adopting the approaches underlying I^3^ theory [27,28] and GST [25] it is likely that for anger and hostility to diminish, changes to the individual’s self-regulation abilities and a decrease in strain brought on by exposure to stressful life events, should occur first. Therefore, the 10 SB-PFT sessions seemed to be the first step in bringing about changes to intra-family relationships and to adolescents’ competence, changes which could lead to reduced anger and hostility in the future.

This study has several limitations. At the methodological level, the results about the inter-group comparison for the evaluation of the effectiveness of the SB-PFT should be taken with caution because of some weaknesses in comparability, such as a significant unequal distribution on the percentage of LSP adolescents in the IG and the CG. Sample specificity has placed restrictions on sample size, preventing us from not only achieving statistically stronger effect sizes, but also from performing additional moderation analyses, which could have led to a better understanding of SB-PFT effectiveness [69]. Moreover, it would have been interesting to conduct a follow-up assessment to identify any changes to the dimensions under study over time, for example, in anger and hostility [69,70]. At the theoretical level, it would be interesting in future studies to assess the presence of stressful life events by considering their permanence throughout the adolescent’s life, and to explore the cumulative effect of stressful life events complementing the effects according to the specific life domain [4]. Moreover, it would be interesting to explore the impact of the stressors not only on the psychosocial adjustment of adolescents, but also on neurological aspects of the adolescent brain [19].

Despite these limitations, this study showed undeniable strengths. The SB-PFT is one of the few group treatments in Spain promoted with public funding from Child Welfare Services, for a very specific population: adolescents with problematic behaviors and their families [51]. Moreover, multiple-family groups promote adolescents to meet other peers in the same situation and provide mutual support [52]. Likewise, this study is one of the first attempts to evaluate the SB-PFT, and to gain an in-depth knowledge of the stressful life events recently experienced by these adolescents. Specifically, studying the impact of stressful life events, according to their nature, contributes to the current approach, which emphasizes the importance of going beyond a cumulative explanation of stressful life events [4]. Finally, the results of this study go beyond the potential effectiveness of the SB-PFT and give us clues about the efficiency of the SB-PFT, according to the stress profiles.

## 5. Conclusions

This research has important implications for the study of psychosocial stress in adolescence, and in working with adolescents with problematic behaviors. Concerning stress profiles, it should be highlighted that: (a) the nature of stress profiles in adolescence is associated with different adjustment characteristics, and therefore different intervention needs; and (b) adolescents with family stressors who also find themselves involved in situations marked by violence, encounter more difficulties in emotional intelligence, exhibit more aggressive behavior, and report less parent attachment than adolescents with only family stressors or a low-stress profile. According to these remarks, from this study it can be concluded that the adolescents’ stress trajectories need to be considered when designing (e.g., groups with similar profiles, deciding on number of sessions); implementing (e.g., objectives and contents tailored to the adolescents’ needs); and assessing (e.g., evaluate dimensions, according to each adolescent stress profile) interventions geared towards adolescents with problematic behaviors.

If we look at the role of stress profiles on SB-PFT effectiveness, the following aspects should be remarked: (a) diversity is seen in SB-PFT effects, according to the nature of the stress profiles, being the adolescents with a family stress profile who benefited more from the treatment, followed by the low-stress profile, and lastly the adolescents with an individual and family stress profile; (b) irrespective of the stress profile, adolescents who participated in SB-PFT improved in some of the emotional intelligence components, being the one clear and shared objective when intervening with any adolescent with problematic behaviors; (c) there are notable changes related to parent attachment in adolescents exhibiting a family- and a low-stress profile; this result is an indicator of how important it is to address family conflict much like SB-PFT does by adopting a multiple-family approach; and (d) SB-PFT should be undertaken alongside other more specific treatments for those adolescents who have been direct victims of violent situations.

It should be noticed that a high drop-out rate was obtained for the group of adolescents participating in the SB-PFT. Abandonment of treatment could be a response of the adolescent to avoid coping with conflictive situations that generate strain [25]. Likewise, the low adolescent attachment with the peer group or therapists may influence the abandonment of SB-PFT. In this framework, to avoid this high drop-out rate, it is essential that professionals work for stablishing therapeutic alliance with the participants [71].

In sum, this study has shown that adolescents with problematic behaviors, living in priority neighborhoods, constitute a heterogeneous group. Those that experience stressors, both at individual and family level, are the most vulnerable to experience maladjustment and require the highest attention from child institutions. The SB-PFT constitutes a valuable treatment for adolescents with problematic behaviors to be considered by CWS, in which emotional intelligence and parent attachment should be the intervention focus, particularly for those adolescents that accumulate family-related stressors.

## Figures and Tables

**Table 1:** Baseline Characteristics for the Intervention Group (IG) and the Control Group (CG): Sociodemographic and Psychosocial Stress Profiles.

Profile	Total (*n* = 210)	IG (*n* = 104)	CG (*n* = 106)	Differences
**Adolescents**				
Girls	50.50%	49.00%	52.00%	χ^2^ = 0.17 *^n.s.^*
Age	*M* = 14.34, *SD* = 1.47	*M* = 14.16, *SD* = 1.48	*M* = 14.52, *SD* = 1.44	*F* = 3.07 *^n.s.^*
**Families**				
Two-parent structure	61.86%	58.33%	65.42%	χ^2^ = 0.86 *^n.s.^*
Number of members	*M* = 4.00, *SD* = 1.10	*M* = 4.07, *SD* = 1.16	*M* = 3.94, *SD* = 1.05	*F* = 0.68 *^n.s.^*
**Family-related stressful events**				
Severe financial problems	46.20%	48.10%	44.30%	χ^2^ = 0.29 *^n.s.^*
Chronic parental conflict	40.00%	47.10%	33.00%	χ^2^ = 4.35 * (0.14) ^1^
Parents’ divorce	28.60%	34.60%	22.60%	χ^2^ = 3.69 *^n.s.^*
Parent’s new partner	24.80%	34.60%	15.10%	χ^2^ = 10.74 ** (0.23) ^1^
Parent’s mental or physical illness	24.30%	30.80%	17.90%	χ^2^ = 4.71 * (0.15) ^1^
**Individual stressful events**				
Bullying (peer victimization)	28.10%	31.70%	24.50%	χ^2^ = 1.35 *^n.s.^*
Victim of intra-family violence	16.70%	21.20%	12.30%	χ^2^ = 2.99 *^n.s.^*
Sexual harassment or abuse	6.70%	8.70%	4.70%	χ^2^ = 1.31 *^n.s.^*

Notes: *n.s.* = non-significant; * *p* < 0.05; ** *p* < 0.01; IG = intervention group; CG = Control group; ^1^ Cramer’s V in brackets.

**Table 2:** Emotional Intelligence, Aggressive Behavior, and Parent Attachment by Stress Profile.

Variables	LSP *M* (*SD*)	FSP *M* (*SD*)	IFSP *M* (*SD*)	Differences	
				*F* (η^2^_partial_)	*DMS*
**Emotional intelligence**					
Intrapersonal	2.59 (0.52)	2.61 (0.67)	2.34 (0.54)	4.14 * (0.04)	LSP-IFSP * FSP-IFSP **
Interpersonal	3.30 (0.49)	3.24 (0.46)	3.26 (0.46)	0.22 *^n.s.^*	-
Adaptability	2.97 (0.62)	3.01 (0.51)	3.01 (0.55)	0.08 *^n.s.^*	-
Stress management	2.57 (0.49)	2.43 (0.63)	2.24 (0.65)	3.74 * (0.04)	LSP-IFSP **
General mood	3.37 (0.56)	3.36 (0.48)	3.03 (0.59)	8.72 *** (0.09)	LSP-IFSP ** FSP-IFSP ***
**Aggressive behavior**					
Anger	2.80 (0.82)	3.09 (0.79)	3.26 (0.85)	3.90 * (0.04)	LSP-IFSP **
Hostility	2.42 (0.71)	2.59 (0.61)	3.01 (0.72)	11.37 *** (0.11)	LSP-IFSP *** FSP-IFSP ***
**Parent attachment**					
Communication	4.02 (0.72)	3.61 (0.93)	3.53 (0.99)	3.72 * (0.04)	LSP-FSP * LSP-IFSP **
Trust	4.19 (0.50)	3.80 (0.83)	3.55 (1.00)	6.89 *** (0.07)	LSP-FSP * LSP-IFSP ***
Alienation	2.05 (0.78)	2.38 (0.82)	2.69 (0.72)	8.36 *** (0.08)	LSP-FSP * LSP-IFSP *** FSP-IFSP *

Notes: *n.s.* = non-significant; * *p* < 0.05; ** *p* < 0.01; *** *p* < 0.001; LSP = low stress profile; FSP = family stress profile; IFSP = individual and family stress profile.

**Table 3:** Scene-Based Psychodramatic Family Therapy (SB-PFT) Effectiveness by Stress Profile.

Variables		IG		CG		LSP	IG		CG		FSP	IG		CG		IFSP
		T1/T2 *M* (*SD*)		T1/T2 *M* (*SD*)		*F* (η^2^_partial_)	T1/T2 *M* (*SD*)		T1/T2 *M* (*SD*)		*F* (η^2^_partial_)	T1/T2 *M* (*SD*)		T1/T2 *M* (*SD*)		*F* (η^2^_partial_)
**Emotional intelligence**	Intrapersonal	2.59 (0.63)	2.65 (0.80)	2.59 (0.50)	2.45 (0.64)	0.84 *^n.s.^*	2.71 (0.63)	2.56 (0.64)	2.50 (0.70)	2.68 (0.64)	3.70 *^n.s.^*	2.24 (0.52)	2.25 (0.58)	2.46 (0.55)	2.34 (0.65)	1.03 *^n.s.^*
	Interpersonal	3.23 (0.65)	3.46 (0.40)	3.32 (0.45)	3.12 (0.51)	4.53 * (0.12)	3.18 (0.45)	3.36 (0.46)	3.30 (0.46)	3.34 (0.41)	2.61 *^n.s.^*	3.23 (0.51)	3.25 (0.45)	3.32 (0.38)	3.28 (0.46)	0.32 *^n.s.^*
	Adaptability	2.88 (0.71)	2.74 (0.85)	3.01 (0.62)	3.06 (0.53)	0.57 *^n.s.^*	3.03 (0.46)	3.26 (0.60)	3.01 (0.56)	2.96 (0.48)	5.72 * (0.06)	2.93 (0.53)	3.06 (0.51)	3.13 (0.56)	3.08 (0.54)	2.94 *^n.s.^*
	Stress management	2.43 (0.27)	2.55 (0.74)	2.62 (0.55)	2.51 (0.53)	1.06 *^n.s.^*	2.30 (0.64)	2.32 (0.76)	2.54 (0.60)	2.60 (0.45)	0.13 *^n.s.^*	2.07 (0.57)	2.13 (0.50)	2.48 (0.70)	2.46 (0.67)	0.35 *^n.s.^*
	General mood	3.33 (0.71)	3.43 (0.53)	3.39 (0.53)	3.27 (0.56)	1.18 *^n.s.^*	3.31 (0.50)	3.38 (0.48)	3.43 (0.47)	3.40 (0.41)	2.00 *^n.s.^*	2.92 (0.59)	3.08 (0.49)	3.17 (0.58)	3.07 (0.63)	5.92 * (0.08)
**Aggressive**	Anger	3.00 (0.94)	2.68 (0.77)	2.74 (0.77)	2.69 (0.87)	0.72 *^n.s.^*	3.18 (0.72)	3.06 (0.95)	3.00 (0.86)	2.89 (0.81)	0.01 *^n.s.^*	3.39 (0.86)	3.33 (0.85)	3.10 (0.81)	3.06 (0.82)	0.01 *^n.s.^*
	Hostility	2.23 (0.81)	3.36 (1.03)	2.52 (0.67)	2.52 (0.77)	0.20 *^n.s.^*	2.54 (0.64)	2.33 (0.68)	2.65 (0.60)	2.59 (0.72)	0.95 *^n.s.^*	2.96 (0.67)	2.89 (0.68)	3.10 (0.77)	2.94 (0.80)	0.12 *^n.s.^*
**Parent attachment**	Communication	3.88 (0.62)	3.62 (0.92)	4.08 (0.76)	3.72 (0.86)	0.08 *^n.s.^*	3.33 (0.98)	3.49 (0.89)	3.91 (0.77)	3.68 (0.82)	5.88 * (0.06)	3.43 (0.97)	3.31 (0.97)	3.74 (0.95)	3.57 (0.92)	0.04 *^n.s.^*
	Trust	4.29 (0.40)	4.08 (0.46)	4.16 (0.54)	3.93 (0.78)	0.01 *^n.s.^*	3.52 (0.90)	3.66 (0.87)	4.09 (0.62)	3.95 (0.71)	4.31 * (0.05)	3.25 (1.04)	3.32 (0.90)	3.99 (0.78)	3.84 (0.80)	2.48 *^n.s.^*
	Alienation	2.35 (1.00)	2.00 (0.68)	1.94 (0.66)	2.33 (0.93)	4.35 * (0.11)	2.62 (0.85)	2.70 (0.86)	2.13 (0.70)	2.23 (0.73)	0.02 *^n.s.^*	2.84 (0.73)	2.92 (0.67)	2.45 (0.67)	2.45 (1.04)	0.19 *^n.s.^*

Notes: *n.s.* = non-significant; * *p* < 0.05; ** *p* < 0.01; *** *p* < 0.001; LSP = low stress profile; FSP = family stress profile; IFSP = individual and family stress profiles.
